# Prevalence of Diabetes Mellitus and Its Risk Factors among Individuals Aged 15 Years and Above in Mizan-Aman Town, Southwest Ethiopia, 2016: A Cross Sectional Study

**DOI:** 10.1155/2018/9317987

**Published:** 2018-04-26

**Authors:** Shiferaw Birhanu Aynalem, Ayalew Jejaw Zeleke

**Affiliations:** ^1^Department of Nursing, College of Health Sciences, Bahir Dar University, Bahir Dar, Ethiopia; ^2^Department of Parasitology, School of Biomedical and Laboratory Sciences, College of Medicine and Health Sciences, University of Gondar, Gondar, Ethiopia

## Abstract

**Introduction:**

Diabetes mellitus (DM), which is related to cardiovascular disease, is one of the main global health problems. In Ethiopia, information about this disease is known to be scarce.

**Objective:**

To assess the prevalence of diabetes mellitus and its risk factors among individuals aged 15 years and above.

**Methods:**

A community-based cross sectional study was carried out from January 01 to March 30, 2016 in Mizan-Aman town, southwest Ethiopia. A multistage sampling technique was used to select study participants. The World Health Organization (WHO) stepwise approach for noncommunicable disease surveillance was deployed to collect data. Total cholesterol and triglyceride level measurements were done using the HumaStar 80 chemistry analyzer. Glucose meter was used to check fasting venous blood glucose level. Descriptive and logistic regression analyses were used.

**Results:**

A total of 402 participants were included in the study. The prevalence of DM was found to be 6.5% (26 out of 402). Of which, the proportion of previously undiagnosed diabetes mellitus was 88.5%. The prevalence of prediabetes was also found to be 15.9%. The waist circumference (WC), body mass index, smoking habit, hypertension, and total cholesterol level were significantly associated with diabetes mellitus.

**Conclusion:**

In this study, higher prevalence of diabetes mellitus was observed than the IDFA-projected estimate of DM for Ethiopia. Modifiable associated risk factors were also identified. Therefore, targeting the prevention strategy to such modifiable risk factors might reduce the prevalence of diabetes mellitus and screening of DM particularly in those individuals having high WC, history of smoking habit, and hypertension needs attention.

## 1. Introduction

Diabetes mellitus (DM) is a metabolic disorder resulting from a defect in insulin secretion, insulin action, or both. Insulin deficiency in turn leads to chronic hyperglycemia with disturbances of carbohydrate, fat, and protein metabolism [1]. It is one of the chronic noncommunicable diseases (CNCDs) which have emerged as a leading global health problem. It is also a known risk factor for blindness, vascular brain diseases, renal failure, and limb amputations [2]. According to the International Diabetes Federation (IDF) Atlas guideline report, currently, there are 352 million adults with impaired glucose tolerance who are at high risk of developing diabetes in the future. In 2017, it was estimated that 425 million people (20–79 years of age) suffered from DM, and the number is expected to rise to 629 million by 2045. Moreover, in 2017, the projected national diabetes (20–79) prevalence in Ethiopia estimated by IDF Atlas was 5.2% [3].

Unfortunately, the trend will continue to exist in low- and middle-income countries despite the misconception that diabetes is “a disease of the well-to-do” [4]. It is estimated that developing countries will bear 77% of the global burden of the DM epidemic in the 21st century [5] as a result of population growth, consumption of unhealthy diets, obesity, and sedentary lifestyles [6].

Ethiopia as one of the developing countries has been showing changes that shifts the lifestyle of the people towards urbanization, particularly in recent decades. These rapid changes have led to the emergence of noncommunicable chronic diseases such as diabetes mellitus. Moreover, due to limited materials and human resources, the major focus of the country is on combating infectious diseases and paying little attention to CNCDs. Assessing the prevalence of DM is important for national health policy planners; therefore, this study is aimed at determining the prevalence of DM and its associated factors among individuals aged 15 years and above in Mizan-Aman town, southwest Ethiopia.

## 2. Methods

### 2.1. Study Setting

The study was conducted in Mizan-Aman town, southwest Ethiopia, 561 km away from Addis Ababa, the capital city of Ethiopia. According to the 2007 national census, the projected total population of Mizan-Aman town is 34,080. Of the total population, 18,138 are males. The town is subdivided into five *kebeles* (the smallest administrative units) with a total number of 8159 households.

### 2.2. Study Design, Period, and Sample Size

A community-based cross sectional study was conducted from January 01 to March 30, 2016. The source population was individuals aged 15 years and above permanently living in the town. The sample size was calculated using the single population proportion formula by considering 5.1% prevalence of DM [7], 0.03 desired precision, 95% confidence interval (CI), and a design effect of 2. Thus, the minimum sample size (*n*) calculated was found to be 414.

### 2.3. Sampling Technique

Multistage sampling technique was employed to select study participants. Of the five *kebeles* in the town, three of them were selected randomly. Then, the sample was allocated to the selected *kebeles* in proportion to the total number of households in each *kebele*. Accordingly, 168, 111, and 135 study participants were sampled from *Sheheka*, *Adis Ketema*, and *Ediget kebeles*, respectively. Households in each *kebele* were selected by the systematic sampling technique by using the list of households as a sampling frame. Finally, if more than one eligible individual were found in a household, a study participant was selected randomly from that particular house.

### 2.4. Exclusion Criteria

- Individuals who were taking any drug with possible impact on glucose metabolism (e.g., steroids, B-blockers, and thiazide diuretics) other than antidiabetes mellitus drugs were excluded to avoid false positive prediabetes or diabetes mellitus.
- Pregnant women were excluded from the study to avoid the possible impact of pregnancy on anthropometric and laboratory parameters.

### 2.5. Data Collection and Measurement

Data on demographic and behavioral characteristics were collected by trained personnel through a face-to-face interview using a semistructured questionnaire. The field study team was composed of enumerators, laboratory technicians, nurses, and supervisors. The World Health Organization (WHO) stepwise approach (three steps) for noncommunicable disease surveillance was used to collect the data [8, 9].

### 2.6. Step 1: Demographic and Behavioral Characteristic Data

In this step, demographic and behavioral risk factors were collected through face-to-face interviews using an interviewer-administered questionnaire. Each participant was questioned for age, sex, educational status, marital status, occupation type, physical activity, history of raised blood pressure and diabetes, fruit and vegetable intake, alcohol consumption, and smoking habit.

### 2.7. Step 2: Physical Measurements

Physical measurements of height and weight needed to calculate body mass index (BMI), waist circumference, and blood pressure were taken in this step. Blood pressure (BP) was taken in a sitting position from the right arm using a digital sphygmomanometer. Two readings were taken 5 minutes apart, and the mean was considered as the final BP result. Prehypertension is defined as systolic BP of 120–139 and diastolic BP 80–89 mmHg. Hypertension is defined as systolic BP of ≥140 mmHg or diastolic BP of ≥90 mmHg. A portable weight and height scale was used to measure the weight of the participant wearing light clothes and height in upright standing position on a flat surface. Then, body mass index (BMI) was calculated by weight in kilograms divided by height in meters squared formula. BMI < 18.5 kg/m^2^ is considered as underweight, 18.5–24.9 kg/m^2^ as normal, 25–29.9 kg/m^2^ as overweight, and ≥30 kg/m^2^ as obese. Waist circumference (WC) was measured at the approximate midpoint between the lower margin of the last palpable rib and the top of the iliac crest, using a flexible plastic tape. WC values > 94 and >80 cm for men and women, respectively, were considered high according to the World Health Organization (WHO) recommendation.

### 2.8. Step 3: Biochemical Measurements

Fasting blood glucose, triglyceride (TG), and total cholesterol level measurements were taken. After an overnight fasting (≥8 h), plasma glucose was determined using the glucose meter Accu-Chek Active system [2, 10, 11]. The Accu-Chek Active system uses a capillary blood sample which is set to plasma serum standard, showing result in plasma glucose values. This measurement was immediately performed for all participants, and the results were recorded in the questionnaire. Fasting capillary blood samples were collected three times at different occasions (for three consecutive days) from a single study participant, and glucose measurement was carried out within fractions of seconds after sample collection. Then, their average was taken for analysis, and this might have minimized the appearance of abnormal results. The diagnosis of DM was based on the American Diabetes Association diabetes mellitus classification criteria with fasting blood glucose of ≥126 mg/dl being considered as positive for DM; impaired fasting glucose, FBG: ≤110 mg/dl to <126 mg/dl; normoglycemic, FBG: ≤61 mg/dl to <110 mg/dl), and hypoglycemic, <61 mg/dl [12].

Moreover, three ml of fasting venous blood was collected from each participant, using EDTA tubes (after an overnight fasting, i.e., ≥8 h) for biochemical measurements. The sample of every participant was taken to Mizan Tepi University Clinical Laboratory for plasma separation. Plasma samples were transferred into 2 ml Eppendorf tubes and stored at −20°C. Finally, all plasma samples were taken to Jimma University Specialized Hospital for total cholesterol (TC) and triglyceride (TG) level determination, using the HumaStar 80 chemistry analyzer (Human Diagnostic, Germany) as previously described [13].

### 2.9. Data Quality Assurance

Data collectors were refreshed on proper measurement and sample collection. Regular field supervisions were carried out to monitor the field work, and data was collected during the actual field data collection period. After blood samples were collected, plasma was separated and placed at −20°C prior to analysis. The instrument, HumaStar 80 chemistry analyzer, was calibrated using a calibrator (AutoCal), and quality control samples normal (HumaTrol N) and pathological (HumaTrol P) were run each day before running samples for tests. The manufacturer's instructions of the machine and the reagents were strictly followed.

### 2.10. Data Analysis

The data was entered, cleaned, and analyzed using the SPSS version 20.0 software package. Descriptive statistics were used to summarize the characteristics of study participants. Bivariate and multivariate analyses were used to assess the association between explanatory variables and the outcome variable. All explanatory variables with *p*-value of ≤0.2 in the bivariate analysis were inserted in the multivariate binary logistic regression model to see the independent effect of each variable on diabetes. The magnitude of the association was measured using the adjusted odds ratio (AOR) and 95% confidence interval (CI). A *p*-value < 0.05 was considered as statistically significant.

### 2.11. Ethical Approval and Consent to Participate

Ethical approval was obtained from the Research Ethics Review Board of Mizan Tepi University. Informed verbal consent was gathered from each participant. Any information obtained in each course of the study was kept confidential. Participants identified with hypoglycemia, impaired fasting glucose (IFG), and diabetes were referred to nearby health facilities for further investigation and management.

## 3. Results

### 3.1. Sociodemographic Variables

A total of 402 participants with a response rate of 97.1% successfully participated in the study. Twelve samples from the total of 414 participants were found insufficient for biochemical analysis and were excluded from the study. The age of the participants ranged from 15 to 78 years with a mean of 31 (SD = ±6.5). The sociodemographic characteristics of the variables were summarized in Table 1.

### 3.2. Behavioral Characteristics

About one third (32.1%) of the total (402) participants said that they were frequent alcohol drinkers, whereas 1% (4/402) of them reported that they were ex-drinkers. Other behavioral characteristics of the study subjects are presented in Table 2.

### 3.3. Physical and Biochemical Measurements

Out of the total study participants, 6.5% of them had ≥126 mg/dl fasting blood glucose level. The different types of physical and biochemical measurements are summarized in Table 3.

### 3.4. Prevalence of Diabetes Mellitus

**Figure 1:**
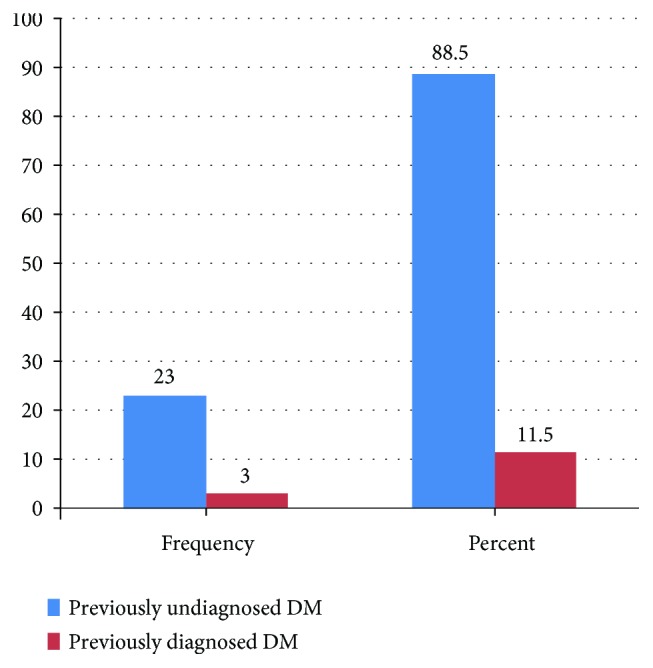
Distribution of previously diagnosed and undiagnosed DM among DM-positive participants, in Mizan-Aman town, southwest Ethiopia, 2016.

The majority, 77.1% (310/402), of the study participants were normoglycemic, whilst 15.9% (64/402) of the respondents were prediabetics (Table 3). The prevalence of DM was found to be 6.5% (26 out of 402). Out of individuals who were found to be diabetic, the proportion of previously undiagnosed DM was 88.5% (23/26) (Figure 1).

### 3.5. Factors Associated with Diabetes Mellitus

Study participants with high waist circumference were 4.1 times more likely to be DM positive compared to those whose waist circumference was normal (AOR = 4.107, 95% CI: 1.108, 15.231). Regarding body mass index, being overweight was also independently associated with the prevalence of DM. Respondents who were overweight were 4.1 times at more risk of being DM positive than those with normal body mass index (AOR = 4.163, 95% CI: 1.516, 11.435). Similarly, individuals with smoking habit were about 27 times more likely to be DM positive when compared to participants who never smoked in their lifetime (AOR = 26.946, 95% CI: 3.146, 230.819) (Table 4).

## 4. Discussion

This study shows that the prevalence of DM is found to be 6.5% (26 out of 402). This finding is comparable to the International Diabetes Federation Atlas-projected estimate of DM for Ethiopia (5.2%), to those of studies done in Gondar Town and Dabat residential districts which together reported 5.11%, and Bishoftu town in which a prevalence of 5% was detected [9, 14]. On the other hand, the result is higher than those of other studies done in Gilgel Gibe (4.4%) and Ayder Referral Hospital (1.3%) [14–16]. This discrepancy may be due to differences in the study area, study designs, sample sizes used, and the times the studies were conducted. For example, the studies conducted at Gilgel Gibe and Ayder Referral Hospital were included both urban and rural areas and huge sample sizes. The present study was conducted on a relatively small sample compared to the sample sizes of the other studies, and it was exclusively done among urban residents. Moreover, urbanization can influence the lifestyles of people in general, and the prevalence of DM among urban dwellers is usually higher than among rural householders. Besides, our result is lower than those reported from Kinshasa [17] and India [18]. The direct comparisons of prevalence rates might be difficult owing to different methodologies and diverse characteristics of the study participants.

The present study revealed that the proportion of undiagnosed DM was 88.5% (Figure 1). This is quite higher than the reports from South Africa [19] and India [20]. A high proportion of newly diagnosed diabetes mellitus indicates a substantial burden of undetected cases in the study community. This is probably due to the low health-seeking behavior since primary health care services are not comprehensive in developing countries unlike those in developed countries [21].

The prevalence of prediabetes in the present study was found to be 15.9%. This is higher than the estimated Ethiopian national prevalence of 6–8% [4] and those in other parts of the country (9.7%) [15]. This suggests that the prevalence of DM in the study area may increase in the near future as there is a risk of progression of prediabetic condition to diabetic [4].

The present study has described some proven and hypothesized associated factors. This study revealed that there was a significant association between smoking habit and diabetes mellitus (smokers were about 27 times more likely to be DM positive), as noted by previous studies [18, 22]. The exact mechanism why smoking increases the risk of diabetes and deteriorates glucose homeostasis has not been fully elucidated, but the available evidence shows that the habit increases insulin resistance. Smoking has also further been associated with the risk of chronic pancreatitis and pancreatic cancer [23].

Total cholesterol (TC) level was also a significant risk factor for diabetes. Participants who had high TC level (≥200 mg/dl) were about 7.4 times more likely to develop diabetes mellitus than their counter parts. Similarly, the prevalence of DM was higher among participants with a high level of triglycerides (TGs). This is in line with the explanation that individuals with elevated levels of total triglycerides as well as raised low-density lipoprotein (LDL) cholesterol levels are at high risk of developing DM and other cardiovascular diseases [24].

Hypertension, which is a silent and invisible killer condition, has been known a long time ago. This chronic condition and other risk factors, such as diabetes mellitus, often appear together [21]. The present study also showed that the prevalence of DM among hypertensive individuals was found to be 17.3%, and the risk of developing DM was about 4.7 times more than among those whose blood pressure was normal. This result is in agreement with other findings from Bishoftu [14], Pakistan [25], and Indonesia [26].

The other variables that showed significant associations in the multivariable analysis were body mass index and waist circumference. Overweight participants were 4.1 times more likely to be DM positive than normal individuals. This is in agreement with the result of studies conducted in India [20] and other parts of Ethiopia [27]. Similarly, a prevalence of 11.2% DM was observed among participants with high-level waist circumference who were 4.1 times more risky for being diabetic than their counterparts. The same findings were reported from Indonesia [26], India [20], and other parts of the Ethiopia [14]. It has been postulated that expanded abdominal fat stores affect insulin action by releasing free fatty acids (FFA). In addition, fat cells secrete signaling factors, for example, interlukein-6 (IL-6) and tumor necrosis factor-*α* (TNF-*α*) which are involved in the development of insulin resistance [28].

The major limitation of this study is that diabetes mellitus was diagnosed by glucose meter from capillary blood; this is not as accurate and reliable as plasma glucose estimation diagnosed using a spectrophotometer/colorimeter. Moreover, it is possible that participants did not trace their exact fasting time and this might have affected the overall DM prevalence. The study was not able to identify the different types of DM, and this is the other limitation of the study.

## 5. Conclusion

This study indicated 6.5% prevalence of diabetes mellitus which was somewhat higher than the projected national prevalence of DM (5.2%) by IDFA. This result is an alarming condition as it has been predicted that much of the global increase in DM is forecasted to be in developing countries including Ethiopia. As the proportion of undiagnosed DM was high, there might be a large number of people who have DM in the study area but are not aware of it. Waist circumferences (WC), body mass index, smoking habit, hypertension, and total cholesterol level were significantly associated with the diabetes. These factors associated with DM were potentially modifiable. Therefore, targeting the prevention strategy to such modifiable risk factors might reduce the prevalence of diabetes mellitus in the area.

## Figures and Tables

**Table 1:** Sociodemographic characteristics of the study population aged 15 years and above in Mizan-Aman town, southwest Ethiopia, 2016.

Variable	Frequency	Percentage
*Sex*		
Male	151	37.6
Female	251	62.4
*Age group (years)*		
15–24	121	30.1
25–34	130	32.3
35–44	65	16.2
45–54	43	10.7
≥55	43	10.7
*Level of education*		
Illiterate	84	20.9
Able to read and write	36	9
Elementary school	123	30.6
High school	95	23.6
Diploma and above	64	15.9
*Occupation*		
Government employee	91	22.6
Nongovernment employee	5	1.2
Merchant	56	13.9
Daily laborer	17	4.2
Student	62	15.4
Housewife	134	33.3
House servant	13	3.2
Retired	10	2.5
Other	14	3.5
*Family monthly income in Ethiopian birr*		
<300	73	18.2
301–600	119	29.6
601–900	54	13.4
901–1200	59	14.7
>1201	97	24.1
*Family history of diabetes mellitus*		
Yes	20	5
No	382	95
*Family history of hypertension*		
Yes	84	20.9
No	318	79.1

**Table 2:** Behavioral characteristics of study population aged 15 years and above at Mizan-Aman town, southwest Ethiopia, 2016.

Variables	Frequency	Percentage
*Alcohol consumption*		
Nondrinker	269	66.9
Frequent drinker	129	32.1
Ex-drinker	4	1
*Smoking habit*		
Nonsmoker	387	96.3
Smoker	9	2.2
Ex-smoker	6	1.5
*Physical activity*		
Sedentary	41	10.2
Moderate	339	84.3
Vigorous	22	5.5
*Oil consumption*		
Liquid oil	6	1.5
Cruddy oil	394	98
No oil used	2	0.5
*Frequency of eating any fruit*		
Every day	199	49.5
Every three day	125	31.1
Once a week	60	14.9
Once a month	14	3.5
Not eating	4	1
*Frequency of eating any vegetables*		
Every day	177	44
Every three day	155	38.6
Once a week	58	14.4
Once a month	12	3
Not eating	0	0
*Frequency of eating fatty meat*		
Every day	5	1.2
Every three day	10	2.5
Once a week	52	12.9
Once a month	145	36.1
Not eating	190	47.3

**Table 3:** Physical and biochemical measurement characteristics of study population aged 15 years and above at Mizan-Aman town, southwest Ethiopia, 2016.

Variables	Frequency	Percentage
*Hypertension*		
Yes	73	18.2 (14.39–21.92)
No	329	81.8 (78.07–85.60)
*Waist circumference*		
Normal	206	51.2 (46.35–56.13)
High	196	48.8 (43.86–53.64)
*Body mass index*		
Underweight	58	14.4 (10.99–17.86)
Normal	245	60.9 (56.43–65.95)
Overweight	82	20.4 (16.45–24.33)
Obese	17	4.2 (2.26–6.19)
*Fasting blood glucose*		
Diabetic	26	6.5 (4.06–8.87)
Prediabetic	64	15.9 (12.34–19.49)
Normoglycemic	310	77.1 (73.00–81.22)
Hypoglycemic	2	0.5 (−0.19–1.18)
*Total cholesterol*		
<200 mg/dl	388	96.5 (94.1–97.93)
≥200 mg/dl	14	3.5 (1.69–5.27)
*Triglyceride*		
<150 mg/dl	358	89.1 (86.00–92.10)
≥150 mg/dl	44	10.9 (7.89–13.99)

**Table 4:** Multivariable analysis of factors associated with diabetes mellitus among peoples aged 15 years and above at Mizan-Aman town, southwest Ethiopia, 2016.

Variable	DM status		COR, 95% CI	AOR, 95% CI
	Yes (%)	No (%)		
*Age*				
15–24	2 (1.7)	119 (98.3)	0.347 (0.069, 1.755)	
25–34	6 (4.6)	124 (95.4)	1	
35–44	6 (9.2)	59 (90.8)	2.102 (0.650, 6.794)	
45–54	4 (9.3)	39 (90.7)	2.120 (0.569, 7.898)	
≥55	8 (18.6)	35 (81.4)	4.724 (1.537, 14.521)	
*Waist circumference*				
Normal	4 (1.9)	202 (98.1)	1	
High	22 (11.2)	174 (88.8)	6.385 (2.159, 18.886)	4.107 (1.108, 15.231)^∗^
*Body mass index*				
Underweight	0 (0)	58 (100)	0.828 (0.177, 3.884)	0.000
Normal	9 (3.7)	236 (95.9)	1	1
Overweight	13 (15.9)	69 (84.1)	4.940 (2.026, 12.045)	4.163 (1.516, 11.435)^∗^
Obese	4 (23.5)	13 (76.5)	8.068 (2.191, 29.707)	3.523 (0.824, 15.055)
*Smoking habit*				
Nonsmoker	24 (6.2)	363 (93.8)	1	1
Smoker	2 (22.2)	7 (77.8)	4.321 (0.851, 21.943)	26.946 (3.146, 230.819)^∗^
Ex-smoker	0 (0)	6 (100)	0.00 (00.00)	0.000
*Frequency of eating fatty meat*				
Not eating	7 (3.7)	183 (96.3)	1	
Every day	1 (20)	4 (80)	6.536 (0.644, 66.351)	
Every three day	2 (20)	8 (80)	6.536 (1.166, 36.629)	
Once a week	6 (11.5)	46 (88.5)	3.410 (1.093, 10.634)	
Once a month	10 (6.9)	135 (93.1)	1.937 (0.719, 5.218)	
*Hypertension*				
Yes	13 (17.8)	60 (82.2)	5.267 (2.327, 11.920)	4.768 (1.899, 11.971)^∗^
No	13 (4)	316 (96)	1	1
*Total cholesterol*				
<200 mg/dl	22 (5.7)	366 (94.3)	1	
≥200 mg/dl	4 (28.6)	10 (71.4)	6.655 (1.932, 22.922)	7.485 (1.728, 32.418)^∗^
*Triglyceride*				
<150 mg/dl	18 (5)	340 (95.0)	1	
≥150 mg/dl	8 (18.2)	36 (81.8)	4.198 (1.705, 10.333)	

COR = crude odds ratio, AOR = adjusted odds ratio. ^∗^Found significant at 0.05 level significance.

## References

[1] Lebovitz H. E. (2000). Diagnosis, classification, and pathogenesis of diabetes mellitus. *The Journal of Clinical Psychiatry*.

[2] Chobanian A. V., Bakris G. L., Black H. R. (2003). The seventh report of the joint national committee on prevention, detection, evaluation, and treatment of high blood pressure: the JNC 7 report. *JAMA*.

[3] Centers for Disease Control and Prevention (2017). *National Diabetes Statistics Report, 2017*.

[4] Aguiree F., Brown A., Cho N. H. (2013). *IDF Diabetes Atlas*.

[5] Nandeshwar S., Jamra V., Pal D. (2010). Indian diabetes risk score for screening of undiagnosed diabetic subjects of Bhopal city. *National Journal of Community Medicine*.

[6] Chi-Shing Cho W., Kin-Man Yue K., Wing-Nang L. A. (2005). An outline of diabetes mellitus and its treatment by traditional Chinese medicine & acupuncture. *Journal of Chinese Medicine*.

[7] Abebe S. M., Berhane Y., Worku A., Assefa A. (2014). Diabetes mellitus in North West Ethiopia: a community based study. *BMC Public Health*.

[8] Riley L., Guthold R., Cowan M. (2016). The World Health Organization STEPwise approach to noncommunicable disease risk-factor surveillance: methods, challenges, and opportunities. *American Journal of Public Health*.

[9] Azizi F., Ghanbarian A., Momenan A. A. (2009). Prevention of non-communicable disease in a population in nutrition transition: Tehran lipid and glucose study phase II. *Trials*.

[10] National Institutes of Health (2000). The practical guide: identification, evaluation, and treatment of overweight and obesity in adults. http://www.nhlbi.nih.gov/guidelines/obesity/prctgd_c.pdf.

[11] World Health Organization (2011). Waist circumference and waist-hip ratio.

[12] American Diabetes Association (2014). Diagnosis and classification of diabetes mellitus. *Diabetes Care*.

[13] Berhane T., Yami A., Alemseged F. (2012). Prevalence of lipodystrophy and metabolic syndrome among HIV positive individuals on highly active anti-retroviral treatment in Jimma, South West Ethiopia. *Pan African Medical Journal*.

[14] Megerssa Y. C., Gebre M. W., Birru S. K., Goshu A. R., Tesfaye D. Y. (2013). Prevalence of undiagnosed diabetes mellitus and its risk factors in selected institutions at Bishoftu Town, East Shoa, Ethiopia. *Journal of Diabetes & Metabolism*.

[15] Seifu W., Woldemichael K., Tsehaineh B. (2015). Prevalence and risk factors for diabetes mellitus and impaired fasting glucose among adults aged 15–64 years in Gilgel Gibe Field Research Center, Southwest Ethiopia, 2013: through a WHO step wise approach. *MOJ Public Health*.

[16] Whiting D. R., Guariguata L., Weil C., Shaw J. (2011). IDF diabetes atlas: global estimates of the prevalence of diabetes for 2011 and 2030. *Diabetes Research and Clinical Practice*.

[17] OnKin J. B., Longo-Mbenza B., Okwe N., Kabangu N. K., Mpandamadi S. D., Wemankoy O. (2008). Prevalence and risk factors of diabetes mellitus in Kinshasa Hinterland. *International Journal Diabetes & Metabolism*.

[18] Kapoor D., Bhardwaj A. K., Kumar D., Raina S. K. (2014). Prevalence of diabetes mellitus and its risk factors among permanently settled tribal individuals in tribal and urban areas in northern state of sub-Himalayan region of India. *International Journal of Chronic Diseases*.

[19] Motala A. A., Esterhuizen T., Gouws E., Pirie F. J., Omar M. A. K. (2008). Diabetes and other disorders of glycemia in a rural South African community: prevalence and associated risk factors. *Diabetes Care*.

[20] Ahmad J., Masoodi M. A., Ashraf M. (2011). Prevalence of diabetes mellitus and its associated risk factors in age group of 20 years and above in Kashmir, India. *Al Ameen Journal of Medical Sciences*.

[21] World Health Organization (2013). *World Health Day 2013: A Global Brief on Hypertension*.

[22] Cho N. H., Chan J. C. N., Jang H. C., Lim S., Kim H. L., Choi S. H. (2009). Cigarette smoking is an independent risk factor for type 2 diabetes: a four-year community-based prospective study. *Clinical Endocrinology*.

[23] Chang S. A. (2012). Smoking and type 2 diabetes mellitus. *Diabetes & Metabolism Journal*.

[24] Alberti K. G. M. M., Zimmet P., Shaw J. (2006). Metabolic syndrome—a new world-wide definition. A consensus statement from the International Diabetes Federation. *Diabetic Medicine*.

[25] Zafar J., Bhatti F., Akhtar N. (2011). Prevalence and risk factors for diabetes mellitus in a selected urban population of a city in Punjab. *Journal of Pakistan Medical Association*.

[26] Pramono L. A., Setiati S., Soewondo P. (2010). Prevalence and predictors of undiagnosed diabetes mellitus in Indonesia. *Age*.

[27] Yemane T., Belachew T., Asaminew B., Befekadu O. (2007). Type II diabetes mellitus in Jimma town, southwest Ethiopia. *Ethiopian Journal of Health Sciences*.

[28] Ekpenyong C. E., Akpan U., Ibu J. O., Nyebuk D. E. (2012). Gender and age specific prevalence and associated risk factors of type 2 diabetes mellitus in Uyo metropolis, South Eastern Nigeria. *Diabetologia Croatica*.

